# Biomedical text readability after hypernym substitution with fine-tuned large language models

**DOI:** 10.1371/journal.pdig.0000489

**Published:** 2024-04-16

**Authors:** Karl Swanson, Shuhan He, Josh Calvano, David Chen, Talar Telvizian, Lawrence Jiang, Paul Chong, Jacob Schwell, Gin Mak, Jarone Lee

**Affiliations:** 1 Department of Medicine–Clinical Informatics, University of California–San Francisco, San Francisco, United States of America; 2 Massachusetts General Hospital and Harvard Medical School, Boston, Massachusetts, United States of America; 3 Department of Anesthesiology and Critical Care, University of New Mexico Hospital, Albuquerque, New Mexico, United States of America; 4 Temerty Faculty of Medicine, University of Toronto, Toronto, Ontario, Canada; 5 Department of Internal Medicine, Main Line Health Lankenau Medical Center, Wynnewood, Pennsylvania, United States of America; 6 Department of Computer Science, Duke University, Durham, North Carolina, United States of America; 7 School of Osteopathic Medicine, Campbell University, Lillington, North Carolina, United States of America; 8 Sidney Kimmel Medical College, Thomas Jefferson University, Philadelphia, Pennsylvania, United States of America; 9 Department of Psychology, Neuroscience & Behaviour, McMaster University, Hamilton, Ontario, Canada; Mayo Clinic Arizona, UNITED STATES

## Abstract

The advent of patient access to complex medical information online has highlighted the need for simplification of biomedical text to improve patient understanding and engagement in taking ownership of their health. However, comprehension of biomedical text remains a difficult task due to the need for domain-specific expertise. We aimed to study the simplification of biomedical text via large language models (LLMs) commonly used for general natural language processing tasks involve text comprehension, summarization, generation, and prediction of new text from prompts. Specifically, we finetuned three variants of large language models to perform substitutions of complex words and word phrases in biomedical text with a related hypernym. The output of the text substitution process using LLMs was evaluated by comparing the pre- and post-substitution texts using four readability metrics and two measures of sentence complexity. A sample of 1,000 biomedical definitions in the National Library of Medicine’s Unified Medical Language System (UMLS) was processed with three LLM approaches, and each showed an improvement in readability and sentence complexity after hypernym substitution. Readability scores were translated from a pre-processed collegiate reading level to a post-processed US high-school level. Comparison between the three LLMs showed that the GPT-J-6b approach had the best improvement in measures of sentence complexity. This study demonstrates the merit of hypernym substitution to improve readability of complex biomedical text for the public and highlights the use case for fine-tuning open-access large language models for biomedical natural language processing.

## Author summary

Patient access to complex medical information online has driven the need for automated methods for text simplification so that patients can understand and make informed health-related decisions. Deep learning models to process biomedical text has shown great promise in understanding, summarizing, and generation of text. Currently, there remains debate over the most optimal deep learning method for biomedical text simplification. Here, we conducted a study to 1) benchmark three fine-tuned open-access large language models for biomedical text simplification and 2) demonstrate the merit of hypernym substitution to improve text readability. We found that each large language model improved measures of readability and sentence complexity after hypernym substitution. We also identified the GPT-J-6B model was consistently the most optimal method for text simplification based on readability and sentence complexity measures. Our study demonstrates the promise of hypernym substitution for biomedical text simplification and the application of fine-tuned open-access large language models for biomedical text processing.

## Introduction

OpenNotes is a national initiative that launched in 2010 with the primary goal to share clinical notes with patients to increase transparency and patient engagement. On April 5, 2021, a federal mandate implemented the bipartisan 21st Century Cures Act specifying that eight types of clinical notes must be made available and free of charge to all patients [1]. Studies thus far highlight the benefits of shared notes including keeping patients better informed, increased patient satisfaction, and clinical outcomes [2–5]. Despite needing additional support to use patient portals, patients with limited health literacy reported reading clinical notes as considerably important in feeling engaged with their care [3,6]. Adding to the difficulty of understanding complex biomedical text in clinical notes, a study found large numbers of nonstandard words, such as acronyms and abbreviations, in clinical documents. About three-quarters of the words used are not covered in the English and Basic Medical English Dictionary, and 66% of the words in clinical texts are not found in the National Library of Medicine’s Unified Medical Language System (UMLS) [7]. Therefore, simply making clinical charts available may not be equivalent to equitable access.

There remains an outstanding need to make domain-specific biomedical terminologies more interpretable and accessible to patients across varying health literacy rates and languages. Online medical translation tools like Google Translate and MediBabble provide opportunities for improving communication across languages [8], but do not provide any direct form of simplification to medical terminology. Without the right accessibility tools, electronic medical record interpretation may exacerbate existing health disparities.

In the current state of biomedical text disambiguation, many computational tools have been created; ranging from traditional programmatic natural language processing (NLP) rules to a host of advanced artificial intelligence (AI) models, most notably pre-trained neural language models (PNLMs). Historically, automated text simplification started as a pre-processing step of natural language processing to extract salient information [9] and label semantic roles of complex texts [10] using rule-based simplification approaches. Previous programmatic approaches have aimed to replace complex words with simpler synonyms [11] as well as improve selection of important sentences in document-level summarization [12,13]. More recently, artificial intelligence approaches have been applied to achieve text simplification in test scenarios using recurrent neural networks [14] and transformer models coupled with reinforcement learning methods [15]. The advent of general-purpose large language models trained on vast corpora of text have shown great promise in achieving conversational competencies and interactivity at near-human levels across diverse fields, including medicine [16]. Repurposing a general-purpose large language model by fine-tuning on a custom dataset can yield a specialized model that is well-suited for task-specific natural language applications. Applied to the task of simplification of biomedical text, the feasibility and efficacy of fine-tuned large language models compared to standard, rule-based approaches requires further benchmarks for pre-clinical validation.

When navigating the literature and novel innovation surrounding medical lexicon simplification, it is evident that providing layman terminology improves patient comprehension and understanding of medical text [17–19]. Semantic methods to design text simplification approaches generally aim to substitute complex words with related synonyms with the same meaning [20–22]. However, substitution of complex words with their associated synonyms may not effectively simplify words in cases where complex, domain-specific terminology in medicine may not have any reasonable synonyms or only have similarly complex synonyms. To address this problem, we hypothesize that text simplification by substitution with a complex word’s hypernym, the superordinate umbrella term that a complex term falls under, can generate more reasonable simplified text that is readable to lay audiences.

Our objectives were to:

1. Design a method of text augmentation via hypernym substitution that retains semantic and syntactic qualities of biomedical definitions post-sentence augmentation.
2. Perform in silico analysis of a method’s ability to improve the readability of biomedical definitions by applying reliability metrics to method results.
3. Discuss the current readability metrics in the setting of biomedical text augmentation.

## Methods

### Dataset

From the Unified Medical Language System (UMLS), we extracted definitions of each term as a target for text simplification. In total there were 395,168 definitions including all languages. Of those definitions, 331,550 were English. We used simple random sampling to obtain 1000 UMLS definitions, excluding the few hundred used in the training data.

### Pipeline

Our approach uses a linguistic property called hypernymy, which denotes a relationship of specificity between two words or word phrases [23]. An example sentence demonstrating text simplification using hypernymy would be: lymphoma is a type of cancer. The hypernym “cancer” is less specific than the hyponym “lymphoma.”

**Fig 1:**
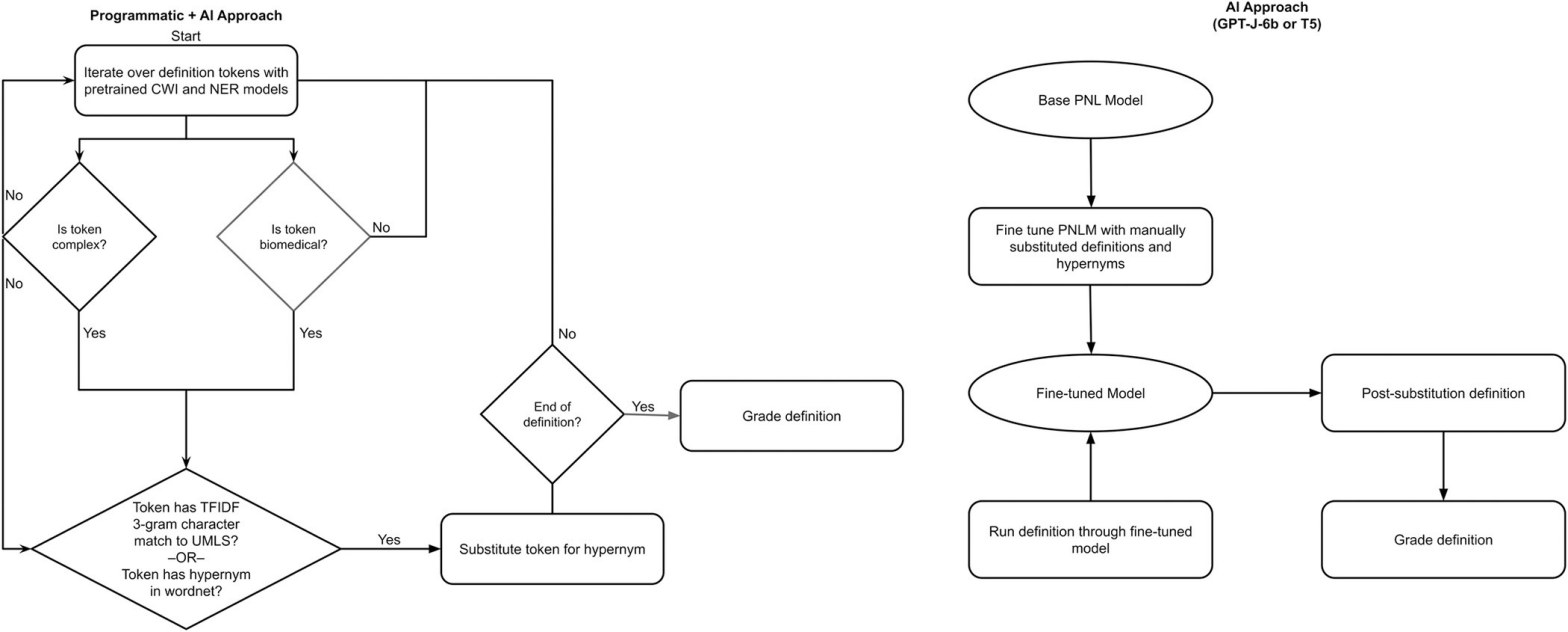
Hypernym substitution process used by three fine-tuned large language model approaches to biomedical text simplification.

The basic approach for hypernym substitution is outlined in **Fig 1**. Complex biomedical words in a given sentence are replaced for their respective hypernym. We show multiple methods for hypernym substitution approaches.

The first approach, named Programmatic + AI, starts with two models: 1) a pre-trained seq2seq bidirectional Long Short Term Memory supervised classification model to identify complex words [24] and 2) a sciBERT large language model to identify biomedical word phrases [25]. If a wordphrase is both complex and biomedical, we then utilize traditional programmatic methods to determine which hypernym replaces the word.

The second approach uses fine-tuned state-of-the-art (SOTA) autoregressive generative transformers, named T5 and GPT-J-6b. We finetuned and used two different model architectures individually to compare performance for the second approach. These models include: 1.) a SciFive T5 model [26] base model architecture, and 2.) a GPT-J-6b [27] base model architecture. The models in the second approach were used to identify which words need substituting and selecting which hypernym would be substituted.

*AI+programmatic approach*: The knowledge base for our corpus was primarily composed of Wordnet and semantic types, with the base node of the hypernym tree coming from the UMLS database. In this approach, we applied the first seq2seq sequence labeling model that determines word complexity [24] and the second sciBERT named-entity recognition (NER) model for the determination of biomedical language substitution [25]. The output of the pre-trained seq2seq model is a prediction of a word’s complexity probability, ranging from 0 to 1. Based on empirical benchmarks of different complexity probability thresholds ranging from 0.1 to 0.9 (0.05 step intervals) inclusive and sample classification outputs of complex words, we set a reasonably balanced threshold of 0.65 based on sensitivity to identify reasonably complex words for model design and further validation. If a word is considered both biomedical and meets the criteria for complexity, with a probability of 0.65 or greater, the word is searched against UMLS with a term-frequency inverse document frequency approach for 3-gram character matching. From there, the matched word’s hypernym root is determined through UMLS semantic types, or from WordNet, whichever has the least amount of syllables.

*Finetuning SciFive T5 and GPT-J-6b*: We utilize two different pre-trained natural language models (PNLM), a base SciFive T5 model and a base GPT-J-6b model^26,27^. The fine-tuning for GPT-J-6b first involved training the model on a Wordnet-derived hypernym dataset of 120,274 hypernymy pairs. We prepared Wordnet-derived hypernym dataset as pairs of words, where each pair consisted of 1) the word to substitute out and 2) the possible hypernym(s) to substitute in and used this dataset to finetune the GPT-J-6b base model using the Hugging Face Auto-Train interface with default parameters. Then, to prime the output fine-tuned GPT-J-6b model to generate hypernym-substituted text at the time of inference, we provided few-shot examples of sentences containing complex words and manually curated output sentences using hypernym substitution based on the Wordnet-derived hypernym dataset. Lastly, we provide the fine-tuned model with input text for automated complex word identification and hypernym substitution. Likewise, we used a similar approach to fine-tune a T5 model using the Hugging Face interface with default parameters. For the T5 model, specifically the SciFive model trained on a PubMed-derived biomedical text corpora, we utilize a fine-tuning training set of manually curated output sentences using hypernym substitution derived from Wikipedia, Simple Wikipedia, and UMLS definitions. None of the manually curated examples in the training set were used in the dataset for evaluation.

### Analysis

After hypernym substitution occurs, we analyze the document’s readability with the Flesch-Kincaid (FK) Reading Ease Score (referred to as FKS) and the respective FK Grade Level scores (referred to as FKG), pre and post-hypernym substitution. Both scores assess sentence lengths (based on word count) and word complexities (based on the number of syllables). The FK Reading Ease is a scale of 0–100, with 100 being the least difficult for reading comprehension. The FKG estimates the United States educational grade level needed for understanding the text. Two other standard reading score methods for comparison included in the model benchmark analysis were the Automated Readability Index (ARI) and Gunning Fog Index (GFI). ARI is an additional estimate of the United States educational grade level needed for comprehending the text. GFI estimates the years of formal education a person needs to understand the text on the first reading. We hypothesized that hypernym substitution for complex-biomedical words increases FKS and therefore decreases the respective FKG level. Similarly, since ARI and GFI are grade measures, we would expect a reduction in the grade level for these measures post-hypernym substitution. The mathematical definitions of FKS, FKG, ARI, and GFI are shown in S1 Fig.

To measure sentence complexity, we compared one measure of lexical diversity and one measure of syntactic diversity between text before and after hypernym substitution using three different large language models. The Measure of Textual Lexical Diversity (MLTD) is an index of the range of different words in a text corpus, where higher scores indicate higher lexical variation. The Mean Dependency Distance (MDD) is an index of text complexity based on the mean linear distance between two syntactically related words in a text. Both measures aim to quantitatively reflect in part the cognitive load of text comprehension beyond standard reading score methods.

To evaluate the human readability of hypernym-substituted text, we conducted a blinded study where two study authors (KS, DC) rated a random sample of original texts and post-hypernym substituted texts (n = 50) based on 5 pilot measures of readability. Texts were rated on a Likert scale scored from 1(very poor) to 5 (very good) for each metric: clarity, conciseness, ease of understanding, accuracy, and fidelity (Table 1). Cohen’s kappa score was used to compare inter-rater reliability. Cohen’s kappa score thresholds for strength of rater agreement ranges from <0 = poor, 0–0.20 = slight, 0.21–0.40 = fair, 0.41–0.60 = moderate, 0.61–0.80 = substantial, and >0.80 = almost perfect [28]. To confirm if hypernym substitution improved human-rated readability, we compared the readability of the original text compared to the readability of the hypernym-substituted texts based on clarity, conciseness, and ease of understanding. Since the original text is considered the gold-standard based on accuracy and fidelity, we did not compare the hypernym-substituted texts to the original texts for these two metrics. To identify the best-performing hypernym substitution approach, we conducted compared each of the hypernym-substituted texts generated using GPT-J-6b, T5, and Programmatic + AI based on clarity, conciseness, ease of understanding, accuracy, and fidelity.

**Table 1:** Definitions of pilot measures of human-rated readability.

Human Readability Metric	Metric Definition
Clarity	Use of terminology and sentence structure to enhance comprehension
Conciseness	Brevity of expression
Ease of Understanding	Accessibility of comprehension without external resources
Accuracy	Preservation of the literary elements of the original text
Fidelity	Preservation of the core interpretation of the original text

## Results

### Dataset

After post-processing through each approach: Programmatic+AI, T5, and GPT-J-6b, some definitions were lost due to model error. We were left with 743 definitions that were successfully processing by all three large language models in the union of these post-processed datasets. Table 2 describes the general characteristics of this composite dataset in terms of readability metric parameters.

**Table 2:** Description of the dataset for definitions to be run through each of the three large language models: Total UMLS Definitions, Total UMLS English definitions, and the UMLS English Subset we used to evaluate our models.

	UMLS All Definitions	UMLS All English Definitions	UMLS Sample English Definitions
**Total # of Definitions**	**394,168**	**331,550**	**1,000**
**Mean Characters Per Definitions**	**262**	**216**	**230**
**Mean Words Per Definition**	**31**	**31**	**31**
**Mean Sentences Per Definition**	**3**	**3**	**3**

### Evaluation

The summary of change for each readability metric for comparison across the three approaches can be seen in the **Table 3**. We additionally compared these approaches’ outputs by calculating the two-tailed Wilcoxon Signed Rank test for the mean readability scores between each approach: *Programmatic+AI* vs *T5*; *Programmatic*+*AI* vs. *GPT-J-6b*, *T5* vs. *GPT-J-6b*. The distribution of FKS, FKG, ARI and GFI scores pre-substitution vs. post-substitution using the Programmatic+AI, T5, and GPT-J-6b models are shown in S1, S2 and S3 Figs respectively.

**Table 3:** Mean change (Δ) in four readability metrics FKS, FKG, ARI, GFI, as well as two sentence complexity metrics MLTD and MDD, of the post-hypernym substituted texts generated using each model compared to the original pre-hypernym substituted texts. P value of two-sided Wilcoxon Signed Rank test are seen below the mean difference comparing between the post-hypernym substituted texts generated using each model and the original pre-hypernym substituted texts (* p < 0.05, ** p < 0.01, *** p < 0.005).

	*Readability Metric*					
*Approach*	*FKS*	*FKG*	*ARI*	*GFI*	*MLTD*	*MDD*
*Programmatic+AI*	Δ32.31***	Δ-4.26***	Δ-4.51***	Δ-4.39***	Δ0.22p = 1.036 x 10^−1^	Δ0.098***
*GPT-J-6b*	Δ31.64***	Δ-4.86***	Δ-10.33***	Δ-3.81***	Δ-25.31***	Δ-0.42***
*T5*	Δ5.63***	Δ-0.89***	Δ-4.41***	Δ-0.51***	Δ0.82p = 4.916 x 10^−1^	Δ-0.22***

Our text simplification approaches were applied to 1000 medical definitions, with a union output of 743 post-processed definitions from the random sample of the UMLS dataset. The post-hypernym substitution output of the GPT-J-6b and Programmatic+AI models reduced grade reading level by around 4 grades, with the exception of the ARI grade, which reduced the reading level by 10 grades using GPT-J-6b approach.

#### Evaluation with FK score

**Fig 2:**
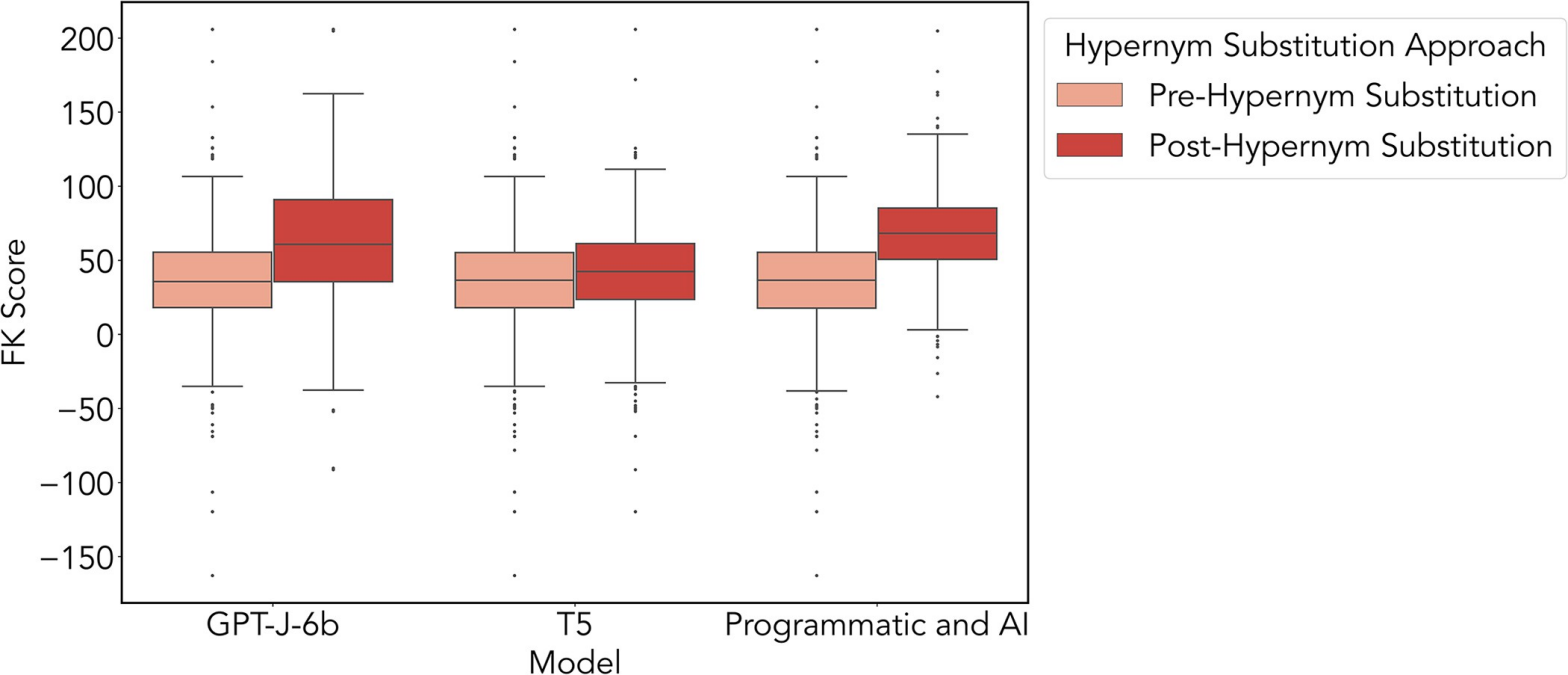
Benchmark of the mean difference in FKS for pre-processing to post-processing for each large language model approach, Programmatic+AI, T5, GPT-J-6b. Here we show that the Programmatic+AI had the most improvement, with a mean FKS increase of 31.67 (p<0.001, IQR 34.58), followed by the GPT-J-6b approach, with a mean FKS increase of 29.65 (p<0.001, IQR 55.38). The least amount of improvement was using the *T5* approach, with a mean FKS increase of 5.67 (p<0.001, IQR 37.66).

The mean difference of post-processed scores were compared across approaches with a two-sided Wilcoxon Signed Rank test. In the Programmatic+AI vs. T5 approaches, the mean score was significantly different (p<0.001, CI = 24.16 to 27.84). In the Programmatic+AI vs. GPT-J-6b approaches, the mean score was not significantly different (p = 3.096x10^-3^, CI = -1.64 to 5.67). In T5 vs. GPT-J-6b approaches, the mean score was significantly different (p<0.001, CI = -27.78 to -20.18). The FK score benchmark test of each large language model before and after hypernym substitution is visualized in Fig 2.

#### Evaluation with FK grade

**Fig 3:**
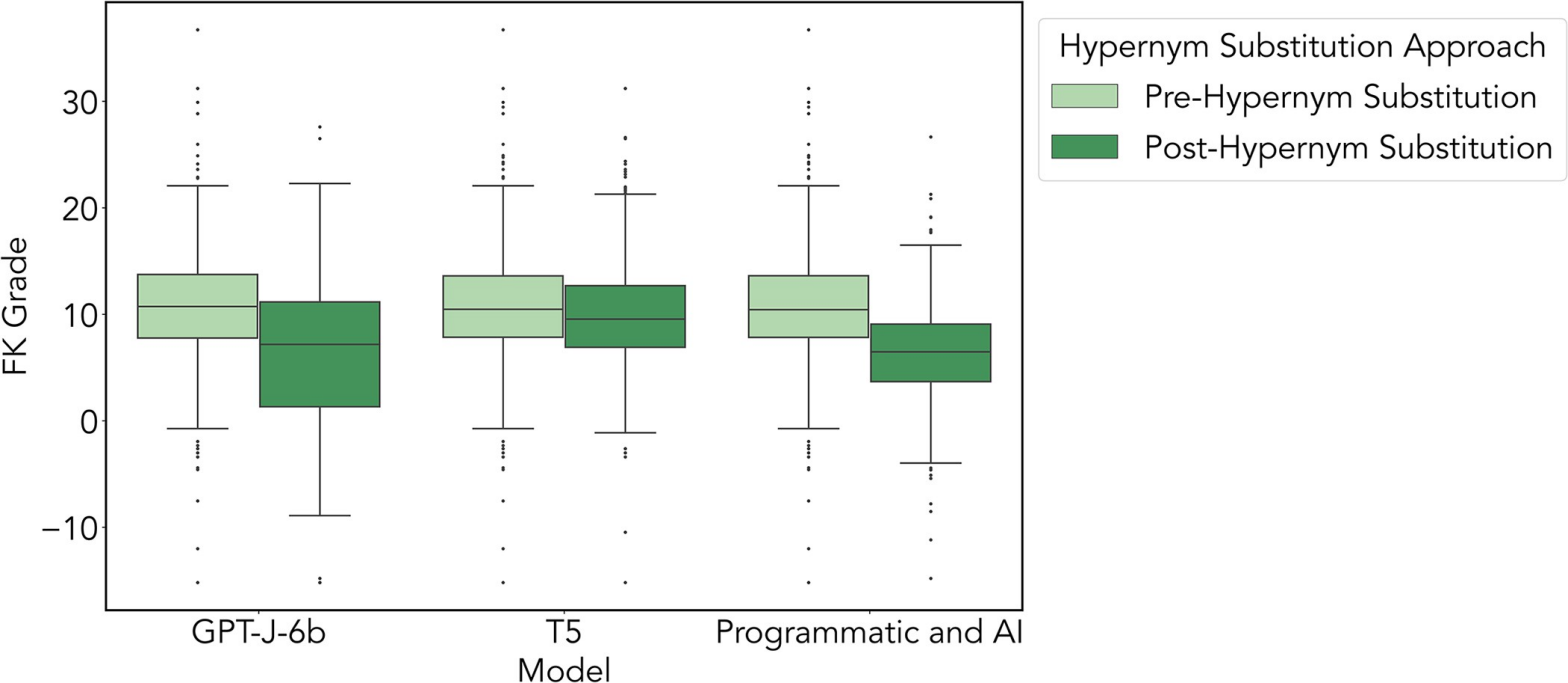
Benchmark of the mean difference in FKG for pre-processing to post-processing for each large language model approach, Programmatic+AI, T5, and GPT-J-6b. Here we show that the Programmatic+AI had the most improvement, with a mean FKG reduction of 4.18 (p<0.001, IQR 5.41), followed by the GPT-J-6b approach, with a mean FKG reduction of 3.25 (p<0.001, IQR 9.84). The least amount of improvement was using the T5 approach, with a mean FKG reduction of 0.94 (p<0.001, IQR 5.77).

The mean difference of post-processed scores were compared across approaches with a two-sided Wilcoxon Signed Rank test. In the Programmatic+AI vs. T5 approaches, the mean difference in FK grade was significantly different (p<0.001, CI = -3.51 to -2.96). In the Programmatic+AI vs. GPT-J-6b approaches, the mean difference in grade was not significantly different (p = 0.297, CI = -1.54 to -0.30). In T5 vs. GPT-J-6b approaches, the mean difference was significantly different (p<0.001, CI = 1.67 to 2.95). The FK grade benchmark test of each large language model before and after hypernym substitution is visualized in Fig 3.

#### Evaluation with ARI

**Fig 4:**
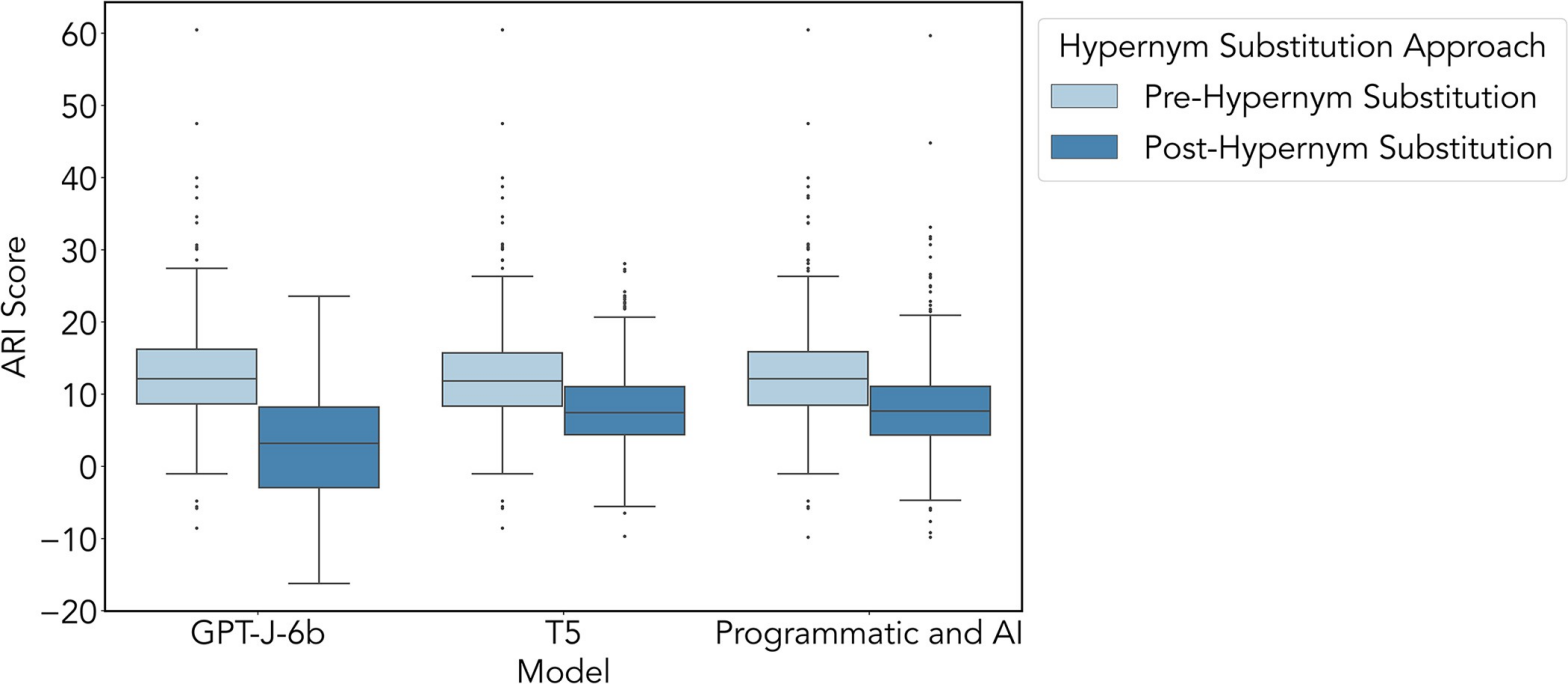
Benchmark of the mean difference in ARI grade for pre-processing to post-processing for each approach, Programmatic+AI, T5, and GPT-J-6b. Here we show that the GPT-J-6b approach had the most improvement, with a mean ARI grade reduction of 10.15 (p<0.001, IQR 11.16), followed by the T5 approach, with a mean ARI grade reduction of 4.70 (p<0.001, IQR 6.65). The least amount of improvement was using the Programmatic+AI approach, with a mean ARI grade reduction of 4.55 (p<0.001, IQR 6.73).

The mean difference of post-processed scores was compared across approaches with a two-sided Wilcoxon Signed Rank test. In the Programmatic+AI vs. T5 approaches, the mean ARI grade was not significantly different (p = 0.676, CI = -0.26 to 0.56). In the Programmatic+AI vs. GPT-J-6b approaches, the mean grade was significantly different (p<0.001, CI = 4.93 to 6.27). In T5 vs. GPT-J-6b approaches, the mean grade was significantly different (p<0.001, CI = 4.82 to 6.09). The ARI grade benchmark test of each large language model before and after hypernym substitution is visualized in Fig 4.

#### Evaluation with GFI

**Fig 5:**
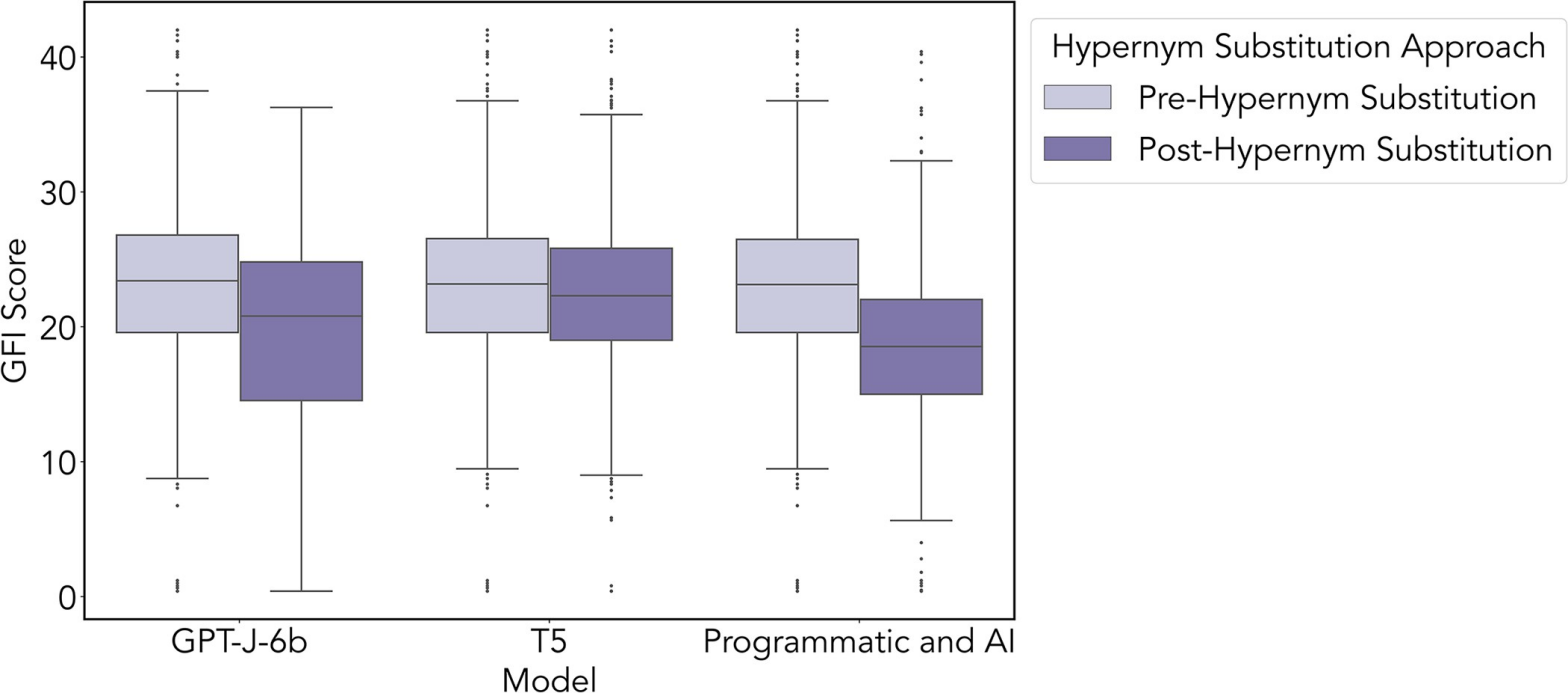
Benchmark of the mean difference in GFI grade for pre-processing to post-processing for each approach, Programmatic+AI, T5, and GPT-J-6b. Here we show that the Programmatic+AI approach had the most improvement, with a mean GFI grade reduction of 4.131 (p<0.001, IQR 7.03), followed by the GPT-J-6b approach, with a mean GFI grade reduction of 3.25 (p<0.001, IQR 6.82). The least amount of improvement was using the *T5* approach, with a mean GFI grade reduction of 0.67 (p<0.001).

The mean difference of post-processed scores was compared across approaches with a two-sided Wilcoxon Signed Rank test. In the Programmatic+AI vs. T5 approaches, the mean GFI grade was significantly different (p<0.001, CI = -4.00 to -3.27). In the Programmatic+AI vs. GPT-J-6b approaches, the mean grade showed a significant difference (p<0.001, CI = -1.69 to -0.41). In T5 vs. GPT-J-6b approaches, the mean grade showed a significant difference (p<0.001, CI = 1.93 to 3.24). The GFI grade benchmark test of each large language model before and after hypernym substitution is visualized in Fig 5.

#### Evaluation with MLTD and MDD

**Fig 6:**
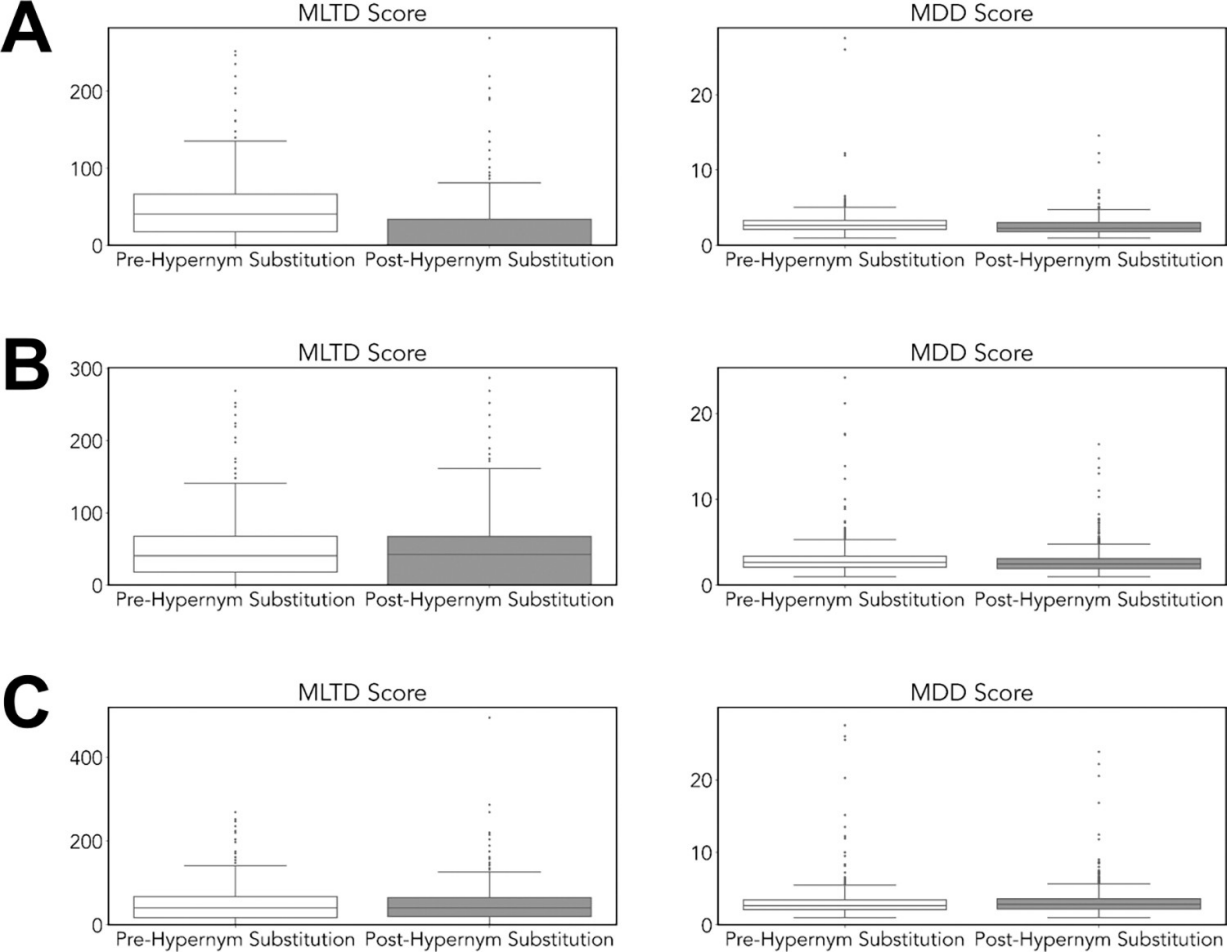
Comparison of the MLTD and MDD scores of text before and after hypernym substitution using the GPT-J-6B, Programmatic+AI, and T5 approaches. A) The GPT-J-6b approach reduced the mean MLTD score from 45.32 to 20.01 (*w* = 10534.5, *p-value* = 3.715x10^-31^) and mean MDD from 2.90 to 2.48 (*w* = 54731, *p-value* = 1.859x10^-11^) between text before and after hypernym substitution. B) The Programmatic+AI approach showed no difference in mean MLTD (*w* = 66876.5, *p-value* = 1.036x10^-1^) and decrease in mean MDD scores (*w* = 94918, *p-value* = 7.519x10^-24^) between text before and after hypernym substitution. C) The T5 approach showed no difference in mean MLTD score (*w* = 93489, *p-value* = 4.916x10^-1^) and reduced the mean MDD score from 2.93 to 2.71 (*w* = 82706.5, *p-value* = 4.227x10^-25^) between text before and after hypernym substitution.

After performing readability metric evaluation, we compared the change in MLTD and MDD metrics, measures of lexical diversity and syntactic complexity respectively. We compared the text before and after hypernym substitution using the GPT-J-6b, programmatic+AI, and T5 approaches. Comparing text before and after hypernym substitution using the GPT-J-6b approach, we observed a decrease in the mean MLTD from 45.32 to 20.01 (*w* = 10534.5, *p-value* = 3.715x10^-46^) and mean MDD from 2.90 to 2.48 (*w* = 4.76, *p-value* = 1.859x10^-11^). Using the programmatic+AI approach, we observed no difference in mean MLTD (*w* = 66876.5, *p-value* = 0.104) but a decrease in mean MDD (*w* = 94918, *p-value* = 7.519x10^-24^) before and after hypernym substitution. For the T5 approach, we observed no difference in mean MLTD before and after hypernym substitution (*w* = 93489, *p-value* = 0.492) and a decrease in mean MDD from 2.93 to 2.71 (*w* = 82706.5, *p-value* = 4.227x10^-25^). The benchmark of the three large language models using MLTD and MDD metrics are visualized in Fig 6.

The mean change in the six readability metrics of the post-hypernym substituted text generated using each of the three models compared to the original pre-hypernym substituted text is summarized in Table 3. Additional results of statistical tests of mean differences in readability scores between pre- and post-hypernym substituted text generated using the Programmatic + AI, T5, and GPT-J-6b approaches are summarized in S1, S2 and S3 Tables respectively.

#### Evaluation with human-rated readability metrics

**Fig 7:**
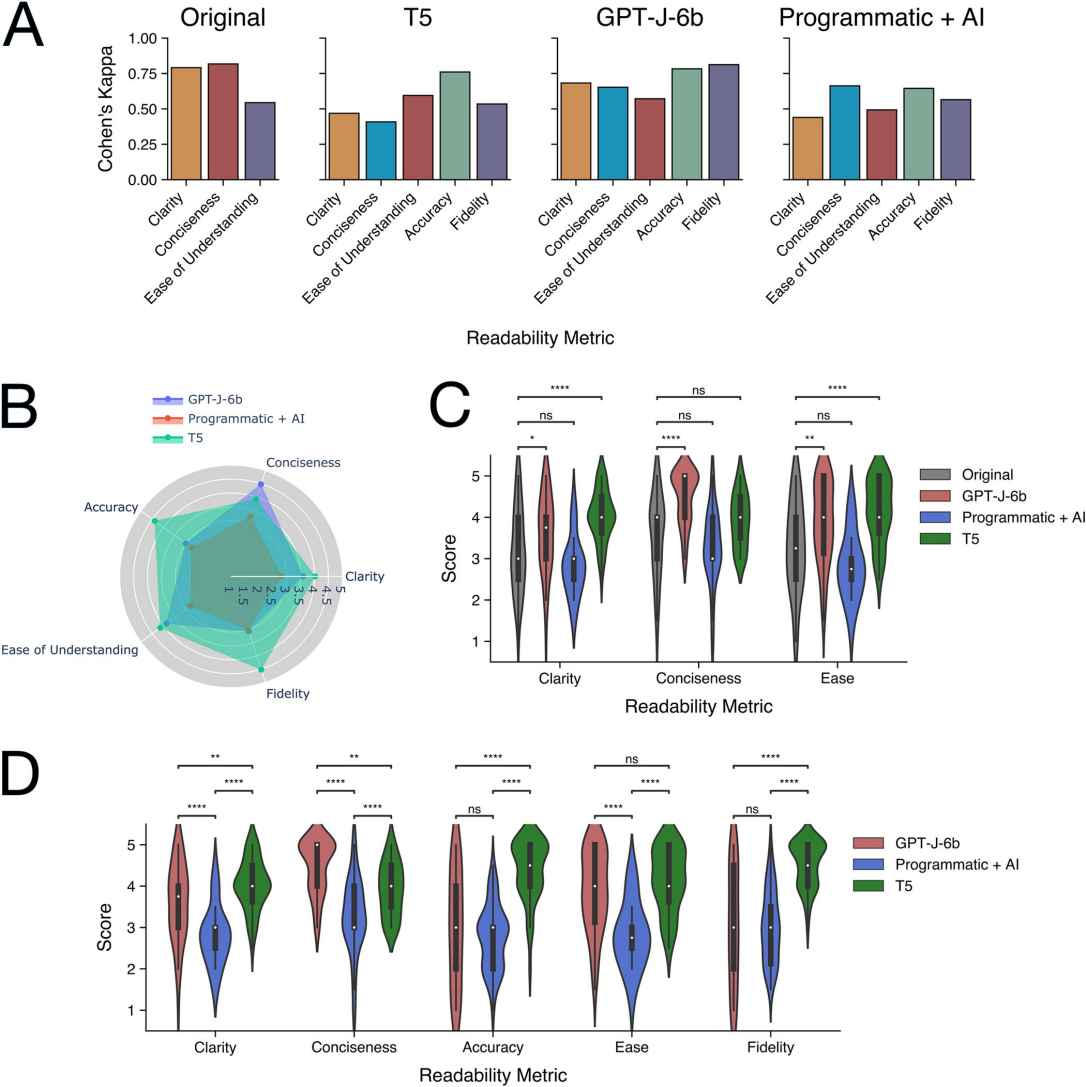
Evaluation of human-rated metrics of text readability after hypernym substitution using the GPT-J-6B, Programmatic+AI, and T5 approaches. A) Cohen’s kappa score across 3 readability metrics of the original text and 5 readability metrics of the hypernym-substituted text. B) C) Comparison of 3 readability metrics between the original text and the hypernym-substituted texts. D) Pair-wise comparison of 5 readability metrics between the hypernym-substituted texts. * = p < 0.05, ** = p < 0.01, *** = p < 0.001, **** = p < 0.0001.

To confirm that the findings of the in-silico readability analyses are represented in human evaluations of text readability, we conducted a blinded study with a random sample of texts (n = 50) where we rated the original text and the 3 hypernym-substituted texts based on pilot measures of human readability. Cohen’s kappa score each of the human-rated readability metrics between two raters confirms moderate to near-perfect inter-rater agreement across all metrics as expected (Fig 7A). Between the 3 hypernym substitution approaches, the T5 approach ranked highest based on mean ratings of accuracy, ease of understanding, fidelity, and clarity while the GPT-J-6b approach ranked highest based on mean ratings of conciseness (Fig 7B). Notably, the Programmatic + AI approach was the lowest-ranked hypernym substitution approach across all five readability metrics.

Next, we compared the readability between the GPT-J-6b, T5, and Programmatic + AI hypernym substituted texts and the original texts to confirm if the hypernym substitution framework improves human-rated readability (Fig 7C; S4 Table). Comparing the GPT-J-6b-generated text to the original text, we observed an increase in clarity (*w* = 138, *p* = 0.0289), conciseness (*w* = 13.5, *p* <0.001), and ease of understanding (*w* = 136, *p* = 0.00105). Likewise, we observed the T5-generated text improved the original text based on clarity (*w* = 21, *p* < 0.001) and ease of understanding (*w* = 36, *p* < 0.001).

To identify the best-performing hypernym substitution approach based on human-rated readability, we conducted pairwise comparisons of readability between the GPT-J-6b, T5, and Programmatic + AI hypernym substituted texts (Fig 7D). The T5 approach ranked the highest based on clarity, accuracy, and fidelity compared to the GPT-J-6b and Programmatic + AI approaches. The GPT-J-6b approach ranked the highest based on conciseness compared to the T5 and Programmatic + AI approaches. The T5 and GPT-J-6B approaches were both ranked higher based on ease of understanding compared to the Programmatic + AI approach. All statistics for inter-approach comparisons of human-rated readability are reported in S5 Table.

## Discussion

### Overview

To the authors’ knowledge, the use of hypernym substitution with fine-tuned open-access large language models, with or without traditional programmatic constraints, for complex medical terminology simplification has not yet been applied for biomedical text. Through our methods, we demonstrated that hypernym substitution shows promise for improving readability of medical and scientific content for the general public. We created two approaches to simplify medical text. The first programmatic+AI approach using deep-learning enabled methods to identify 1) which words or phrases need to be substituted, and then 2) a traditional programmatic rule-based substitution method. The second approach was a one-shot deep-learning approach that handled both the identification of what words or phrases to get substituted and then hypernym substitution itself. The second approach benchmarked two commonly used large language models for the same text simplification task. Both one-shot deep-learning showed improvement across a selection of readability metrics for *in silico* validation. We showed an increase in FKS and a decrease in all grade-level readability metrics for all post-substitution datasets, with the exception of mean GFI score for the T5 approach.

To textually represent our fine-tuned GPT-J-b model, we have provided examples to demonstrate the change from the original UMLS definition to our post-processed definition with hypernym substitution, shown in Table 4. The Programmatic+AI approach, despite post-processing rules to account for grammar and syntax, had results that were qualitatively nonsensical to the authors, despite an improvement in metrics, as demonstrated in Table 4. The GPT-J-6b model did not seem to have these types of erroneous and nonsensical outputs, retaining human-readable syntax and comprehension. This demonstrates that the GPT-J-6b may have better retention of syntax and grammar alongside the measurable reduction in grade level scores per the metrics used compared to programmatic, rule-based approaches. This result is shown quantitatively in the improvement in MLTD and MDD scores, measures of syntaxic complexity, for each hypernym substitution approach. The Programmatic+AI approach did not have a significant difference in these measures between pre and post-hypernym substitution, suggesting no change in cognitive burden. In comparison, the GPT-J-6b approach had a significant reduction in mean MLTD scores, suggesting a net decrease in lexical diversity. Moreover, the GPT-J-6b approach had a significant reduction in syntactic distance, suggesting reduced sentence complexity while overall conserving syntactic structure.

**Table 4:** Example of definitions before and after hypernym substitution for each large language model approach (Programmatic+AI, T5, and GPT-J-6b).

	***Pre-processed Example Sentence***	***FK Grade (rounded)***
	Interacting selectively and non-covalently with a h4 histamine receptor.	14th grade
***Approach***	***Post-processed Example Sentence***	
*Programmatic+AI*	Act selectively and non-covalently with an h4 amine organ.	1st grade
*T5*	Interacting selecting and not binding with a h4 receptor.	9th grade
*GPT-J-6b*	Specifically binding to a histamine h4 receptor.	9th grade

Classic readability formulas may fail to capture linguistic features useful for faithful assessment of human readability [29]. To extend our study beyond in-silico measures of readability, we conducted a cross-sectional study of human-rated evaluations of readability to 1) confirm that the hypernym substitution framework can improve the readability of biomedical text and 2) identify the top-performing hypernym substitution approach. Both the GPT-J-6b and T5 approaches improved clarity and ease of understanding compared to the original text, while only the GPT-J-6b approach improved conciseness compared to the original text. Indeed, these results suggest that the hypernym substitution framework using the GPT-J-6b and T5 approaches can improve the readability of complex domain-specific terminologies within biomedical text. The Programmatic + AI approach failed to improve human-rated measures of readability compared to the original text. Our impression based on the benchmark results was that the programmatic approach failed to capture the appropriate context of the complex biomedical term and failed to retain the structure and syntax of the text, leading to poor accuracy and ease of understanding respectively. We noted that although the GPT-J-6b and T5 approaches improved clarity and ease of understanding compared to the original text, the T5 approach was superior to the GPT-J-6b approach based on accuracy and fidelity to represent the meaning and structure of the original text. The GPT-J-6b approach tended to generate significantly shorter simplified texts than the T5 approach and the original text, leading to loss of important textual elements and meaning in favor of conciseness. Given that optimal text simplification involves both improving readability and retaining the information context of the original text [30], our results demonstrate the superiority of the T5 approach based on its improvement in readability compared to the original text as well as superior performance in readability compared to the GPT-J-6b and Programmatic + AI approaches benchmarked in this study.

Taken together, our results suggest that the T5 approach and its underlying transfer learning framework can be useful for hypernym substitution to improve the readability of complex domain-specific terminology in biomedical texts. The T5 approach is unique compared to the other tested approaches due to its 1) text-to-text framework that directly takes in and outputs text, and 2) large pre-training text corpora on the C4 dataset that is twice as large as Wikipedia. As a framework, transfer learning remains a widely applicable and growing area of machine learning that transfers knowledge learned from one domain to other related source domains [31].

Fine-tuned transformer models remain a promising mode of future research given their ability to be applied to a wide range of natural language processing scenarios, including generative, classification, and regression tasks [32].

### Practical application

For each approach, Programmatic+AI, T5, and GPT-J-6b respectively saw a reduction of FKG by 4.30 (p<0.001), 0.89 (p<0.001), 4.86 (p<0.001). The GPT-J-6b approach had the best results overall for improving readability metrics post-hypernym substitution. In practice, the improvement in readability scores demonstrate successful translation of pre-processed medical lexicon from a collegiate level to a post-processed level of US middle school to high school readability.

OpenNotes mandated that by October 2022, all third-party health applications were required to make personal medical documentation openly available to the public^1^. This increased access allows patients to easily obtain records that were not previously available. With an average of 36% of the United States having a health literacy scored as low [33], and the average reading grade level of American’s is between 7th and 8th grade [34], we anticipate that patients will be looking for a reliable source for understandable medical terminology within the provided context. This process for text simplification using fine-tuned large language models is one proposed solution for addressing biomedical literacy disparities.

### Future development

#### Acronyms and abbreviations

A recent sophisticated and promising NLP model for defining medical acronyms and abbreviations was developed and proved to have high accuracy, but was hindered for operational and production environments by training times [35]. In agreement with Skreta et al., creating a model to handle abbreviations and acronyms is necessary to make biomedical text simplification more readable and understandable to the layperson [35]. However, resolving abbreviations and acronyms alone is likely not sufficient to fully handle the complexity of medical literature and domain-specific terminology. After handling acronyms and abbreviations, hypernym substitution use cases could be used to create simplifying pipelines in other biomedical and clinical contexts, such as clinical text disambiguation. In addition, this process should be optimized for minimal training times.

#### Fine-tuning of large language models

The reduction in readability level from the T5 model was not as significant as the GPT-J-6b model. Future studies adjusting the temperature, defined as the stochasticity of hypernym substitutions and the stringency to perform substitutions as modifiable, is possible. That is, if the temperature was less deterministic and more substitutions were performed, a similar decrease in grade level would be observed for T5 as it was for GPT-J-6b.

Additionally, it remains important that regardless of the approach used an iterative process for a human-in-the-loop system for training is necessary to ensure that translations are working effectively and that corpora data remains valid. We believe a supervised NLP model composed of scientists and medical professionals for model feedback is necessary when handling this specialized jargon used in biomedical text. This would help focus efforts on creating centralized and dependable training data within this domain of research.

#### Open-access large language models

The advent of large language models trained on a vast corpus of natural language serves as a promising next step to build a highly generalizable tool for a variety of natural language processing tasks. For instance, the development of proprietary tools such as ChatGPT, a large language model with over 175 billion parameters, is a promising next step towards building natural language processing. Further fine-tuning of a general large language model such as ChatGPT can be a promising future direction to address the problem of biomedical text simplification. However, there remains promise for the development of alternative open-access large language models, including the models used in this study, so that the process of biomedical text simplification remains transparent, community-sourced, expert-validated, and verifiable by the public.

### Validation

#### Scoring systems

While readability scores have been shown to correlate well with human validation, they are not perfect metrics. Readability formulas have variability up to 6 reading grade levels on the same text [36]. The SMOG (Simple Measure of Gobbledygook) formula was found to perform most consistently and measure the grade level higher, which is why it has been recommended for healthcare applications [37]. Additionally, the NIH identifies the SMOG for the assessment of patient education materials for people with low health literacy [38]. However, SMOG conversion tables for the texts used in this study, with an average length of three sentences would statistically invalid, because the SMOG formula was intended for longer texts and normalized on 30-sentence samples. For our use-case, definitions were on average about 3 sentences long, limiting the utility of this metric. Applying SMOG to future studies looking at large bodies of medical text, such as medical journal publications or portions of a patient chart, could hold potential.

#### Human validation of models

Readability metrics do not inherently account for grammar and syntax. The metrics assume grammar and syntax are intact and sensible, which may not be the case as in the Programmatic+AI approach. That is, an improvement, or reduction, in readability grade does not guarantee an output that is sensical to humans. While the each of the hypernym substitution approaches showed significant differences in terms of grade metric reduction, the authors believe that the T5 and GPT-J-6b approach holds the most promise for a human-readable output **(Table 3)**. Our cross-sectional study of human-rated readability across the 3 hypernym substitution approaches nominated the T5 approach as the top-performing framework for biomedical text simplification using hypernym substitution. Further prospective research is needed to systematically characterize the unique linguistic features of the T5 approach and evaluate patient-based ratings of simplified text.

## Conclusion

We demonstrated that three state of the art large language models can use hypernym substitution to improve readability and decrease semantic complexity useful for biomedical text simplification. We additionally highlight further areas of research needed to validate these proposed models to successfully disambiguate biomedical text.

## Supporting information

S1 Fig Equations used to calculate the readability metrics used to evaluate the Programmatic, T5, and GPT approaches for hypernym substitution of text. (TIF)

S2 Fig Programmatic Approach Distributions of Pre and Post Processing Readability Scores. In this figure we show the distributions of FKS, FKG, GFI, and ARI; red = distributions of original pre-substitution definitions, green = distributions of post-substitution definitions. These figures show change in simplicity distribution for: **A)** FKS—*increase* in score is considered increase in readability. **B)** FKG contrasting the logic of FKS where a *decrease* in grade level is considered and increase in readability. **C) GFI** grade distributions, a decrease in grade level is considered and increase in readability. **D) ARI** grade, a decrease in grade level is considered an increase in readability. Here we show with the programmatic approach, we increase readability across all metrics.
(TIF)

S3 Fig T5 Approach Distributions of Pre and Post Processing Readability Scores. We show the distribution for the **A)** Flesh-Kincaid score, **B)** Flesh-Kincaid grade level, **C)** Gunning Fog grade, and **D)** Automated Readability index for the T5 approach. Score distribution of the pre-substitution text and post-T5 substitution text is shown in red and green respectively. The T5 approach showed an increase in readability across all readability metrics.
(TIF)

S4 Fig GPT Approach Distributions of Pre and Post Processing Readability Scores. We show the distribution for the **A)** Flesh-Kincaid score, **B)** Flesh-Kincaid grade level, **C)** Gunning Fog grade, and **D)** Automated Readability index for the GPT approach. Score distribution of the pre-substitution text and post-GPT substitution text is shown in red and green respectively. The GPT approach showed an increase in readability across all readability metrics.
(TIF)

S1 Table Readability score benchmark of the pre- and post-hypernym substituted text using the Programmatic+AI approach for text simplification. (XLSX)

S2 Table Readability score benchmark of the pre- and post-hypernym substituted text using the T5 approach for text simplification. (XLSX)

S3 Table Readability score benchmark of the pre- and post-hypernym substituted text using the GPT-J-6B approach for text simplification. (XLSX)

S4 Table Human-rated benchmark of 3 readability metrics (clarity, conciseness, ease of understanding) between pre- and post-hypernym substituted text generated using the Programmatic+AI, T5, and GPT-J-6B approaches. (XLSX)

S5 Table Human-rated benchmark of 5 readability metrics (clarity, conciseness, ease of understanding, accuracy, fidelity) between the post-hypernym substituted text generated using the Programmatic+AI, T5, and GPT-J-6B approaches. (XLSX)
