# Supplementary figures and images for: Biomedical text readability after hypernym substitution with fine-tuned large language models
Source: PLOS Digit Health. 2024 Apr 16;3(4):e0000489. doi: 10.1371/journal.pdig.0000489 (PMC11020904; doi:10.1371/journal.pdig.0000489)

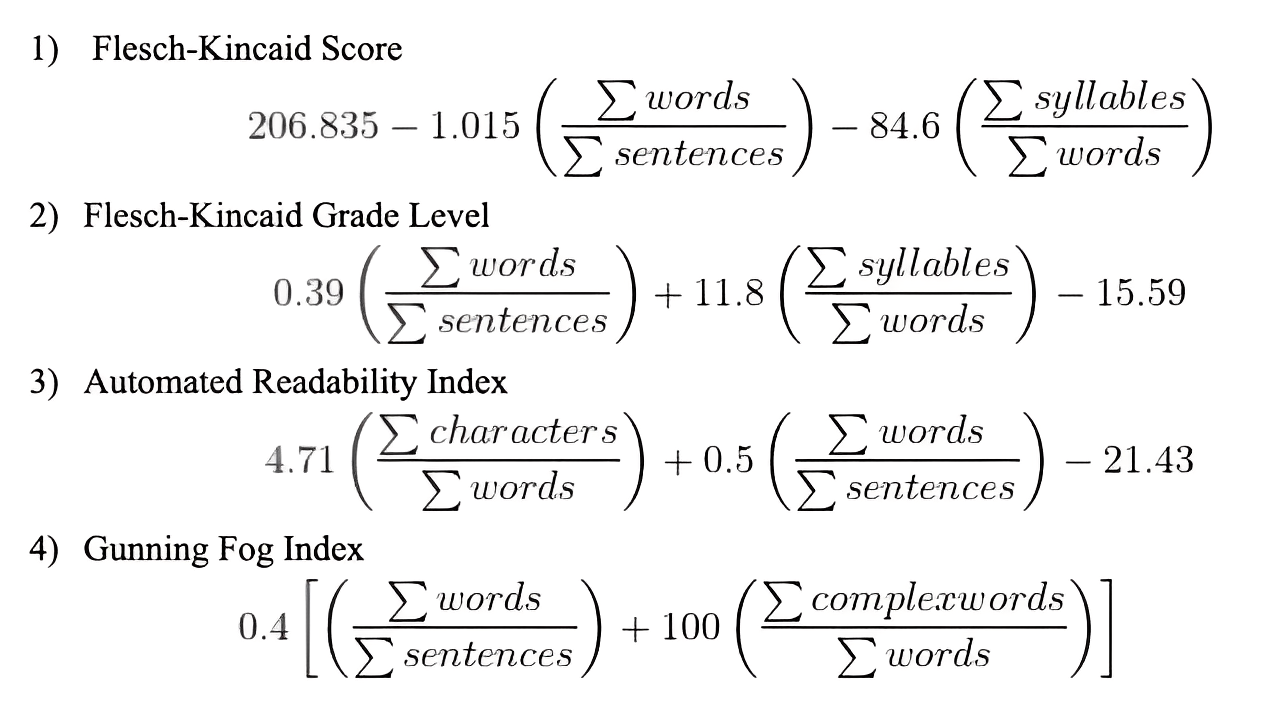

Supplement: S1 Fig — (TIF) [file pdig.0000489.s001.tif]

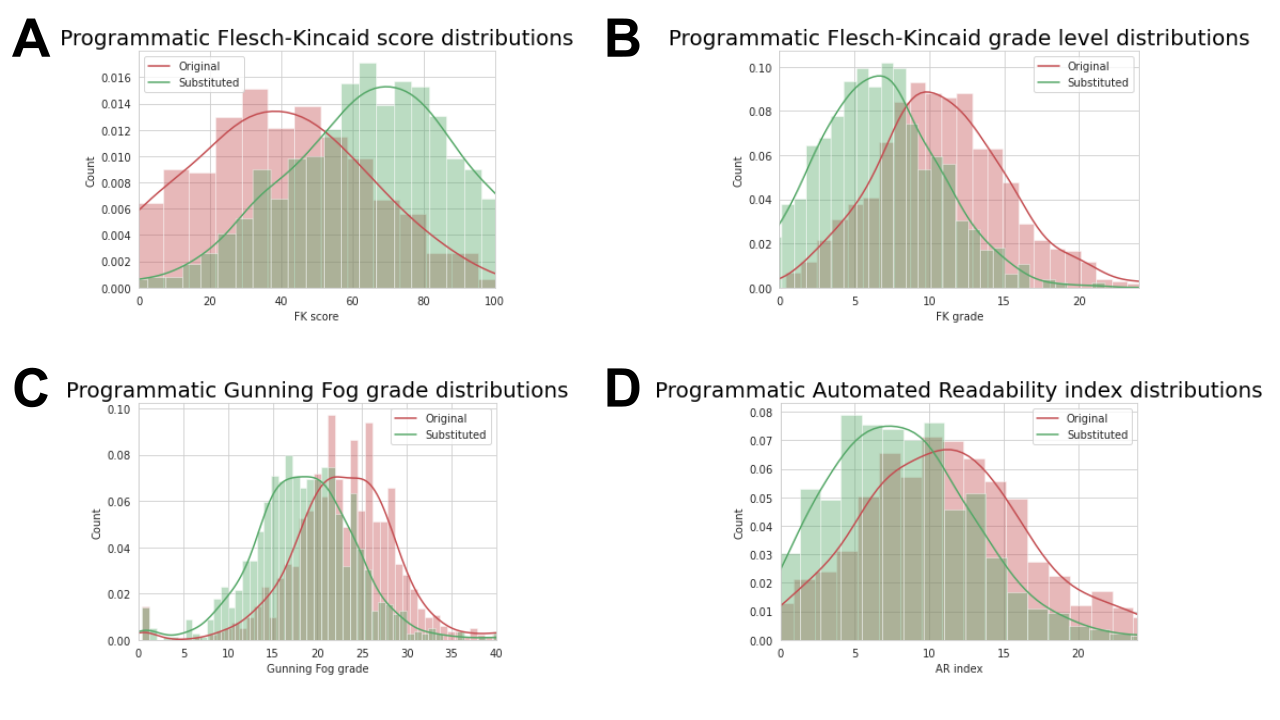

Supplement: S2 Fig — In this figure we show the distributions of FKS, FKG, GFI, and ARI; red = distributions of original pre-substitution definitions, green = distributions of post-substitution definitions. These figures show change in simplicity distribution for: A) FKS—increase in score is considered increase in readability. B) FKG contrasting the logic of FKS where a decrease in grade level is considered and increase in readability. C) GFI grade distributions, a decrease in grade level is considered and increase in readability. D) ARI grade, a decrease in grade level is considered an increase in readability. Here we show with the programmatic approach, we increase readability across all metrics. (TIF) [file pdig.0000489.s002.tif]

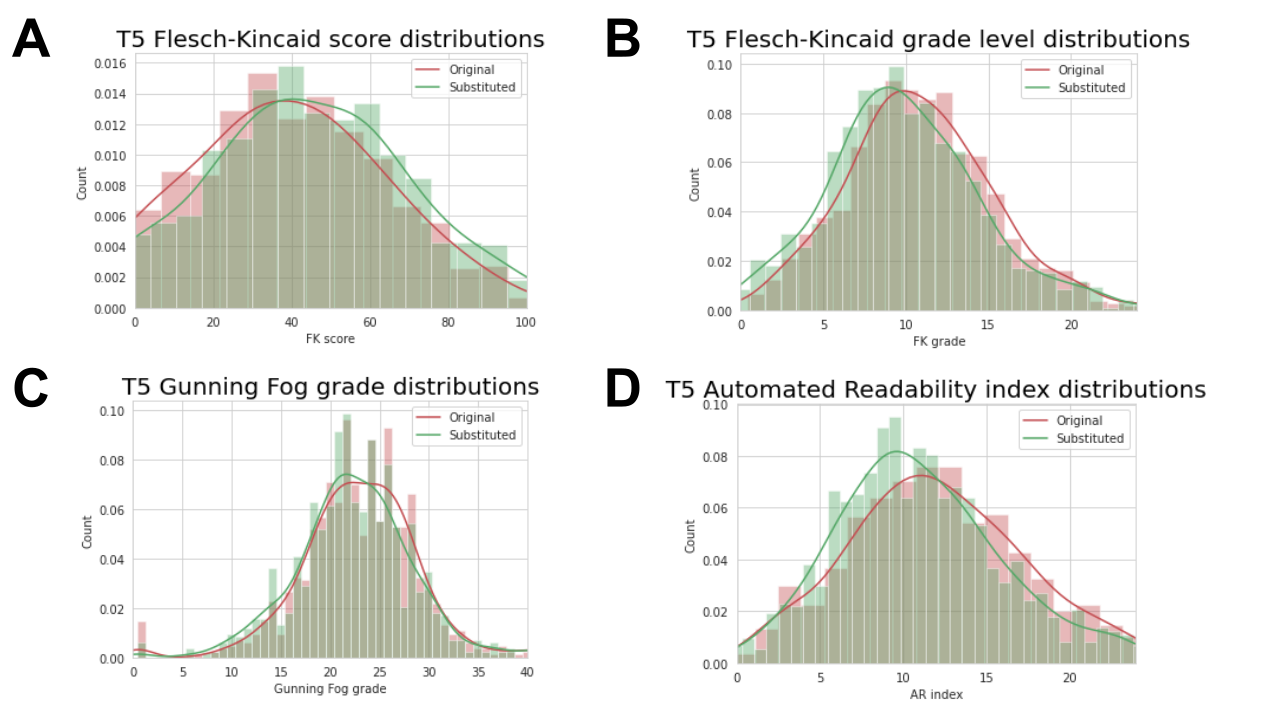

Supplement: S3 Fig — We show the distribution for the A) Flesh-Kincaid score, B) Flesh-Kincaid grade level, C) Gunning Fog grade, and D) Automated Readability index for the T5 approach. Score distribution of the pre-substitution text and post-T5 substitution text is shown in red and green respectively. The T5 approach showed an increase in readability across all readability metrics. (TIF) [file pdig.0000489.s003.tif]

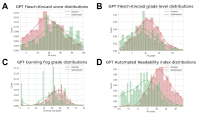

Supplement: S4 Fig — We show the distribution for the A) Flesh-Kincaid score, B) Flesh-Kincaid grade level, C) Gunning Fog grade, and D) Automated Readability index for the GPT approach. Score distribution of the pre-substitution text and post-GPT substitution text is shown in red and green respectively. The GPT approach showed an increase in readability across all readability metrics. (TIF) [file pdig.0000489.s004.tif]
